# Analysis of patterns of livestock movements in the Cattle Corridor of Uganda for risk-based surveillance of infectious diseases

**DOI:** 10.3389/fvets.2023.1095293

**Published:** 2023-01-23

**Authors:** Emmanuel Hasahya, Krishna Thakur, Michel M. Dione, Susan D. Kerfua, Israel Mugezi, Hu Suk Lee

**Affiliations:** ^1^International Livestock Research Institute (ILRI), Kampala, Uganda; ^2^Department of Health Management, Atlantic Veterinary College, University of Prince Edward Island, Charlottetown, PE, Canada; ^3^International Livestock Research Institute (ILRI), Dakar, Senegal; ^4^National Livestock Resources Research Institute (NaLIRRI), Kampala, Uganda; ^5^Department of Animal Health, Ministry of Agriculture, Animal Industry and Fisheries (MAAIF), Kampala, Uganda; ^6^International Livestock Research Institute (ILRI), Hanoi, Vietnam; ^7^College of Veterinary Medicine, Chungnam National University, Daejeon, Republic of Korea

**Keywords:** Uganda, animal movement, network analysis, surveillance system, epidemiology

## Abstract

**Introduction:**

The knowledge of animal movements is key to formulating strategic animal disease control policies and carrying out targeted surveillance. This study describes the characteristics of district-level cattle, small ruminant, and pig trade networks in the Cattle Corridor of Uganda between 2019 and 2021.

**Methodology:**

The data for the study was extracted from 7,043 animal movement permits (AMPs) obtained from the Ministry of Agriculture, Animal Industry and Fisheries (MAAIF) of Uganda. Most of the data was on cattle (87.2%), followed by small ruminants (11.2%) and pigs (1.6%). Two types of networks representing animal shipments between districts were created for each species based on monthly (n = 30) and seasonal (n = 10) temporal windows. Measures of centrality and cohesiveness were computed for all the temporal windows and our analysis identified the most central districts in the networks.

**Results:**

The median in-degree for monthly networks ranged from 0–3 for cattle, 0–1 for small ruminants and 0–1 for pigs. The highest median out-degrees for cattle, small ruminant and pig monthly networks were observed in Lira, Oyam and Butambala districts, respectively. Unlike the pig networks, the cattle and small ruminant networks were found to be of small-world and free-scale topologies.

**Discussion:**

The cattle and small ruminant trade movement networks were also found to be highly connected, which could facilitate quick spread of infectious animal diseases across these networks. The findings from this study highlighted the significance of characterizing animal movement networks to inform surveillance, early detection, and subsequent control of infectious animal disease outbreaks.

## 1. Introduction

The Cattle Corridor covers about 35% of Uganda's land surface and diagonally stretches from southwestern to northeastern Uganda, with many semi-arid characteristics such as; low and unreliable rainfall, and prolonged drought dominated by pastoral rangelands (1, 2). The region has in the present past experienced numerous outbreaks of foot-and-mouth disease (FMD), lumpy skin disease, contagious bovine pleuro-pneumonia in cattle; *peste des petits ruminants*, contagious caprine pleuro-pneumonia in small ruminants; African swine fever in pigs; trypanosomiasis, brucellosis and anthrax in all ruminants and pigs which has partly been fueled by direct animal movement (3–15).

Direct animal movement through animal trade is a major risk factor for the spread of infectious diseases in animals where adequate biosecurity practices and risk management protocols are not followed or are poorly implemented especially in sub-Saharan Africa (16, 17). For example, the spread of the 2001 foot-and-mouth disease (FMD) epidemic from one part of United Kingdom (UK) to geographically distant regions was facilitated by the movement of animals (18).

Therefore, failure to understand animal movement hinders formulation of specific control strategies in case of infectious animal disease outbreaks (19, 20). The lack of animal movement data in Uganda has made it difficult to quantify key parameters for simulating potential disease transmission and hindered effective planning of control strategies for eradication of transboundary animal diseases (TADs) (15, 21).

Uganda has no formal centralized system for identification and traceability of livestock during movement (22). However, a health certificate (commonly known as animal movement permit; AMP) issued by the district veterinary officer (DVO) is required to move animals between districts and even between countries (23). Therefore, the exploration of data from AMPs can help veterinary epidemiologists in Uganda to understand previous outbreaks, predict epidemic spread, and guide decision-making as far as disease control and prevention in livestock are concerned (24). Network analysis is a useful tool that can be used to evaluate different forms of contact between different points/nodes (such as farms, markets, villages, and districts) in the livestock trade and their frequency, as well as how they may play a potential role in the spread of infectious diseases between animal populations (24–26). There is a correlation between the connectivity and centrality of a node within a network, such as the number of other nodes to which it is linked, with the probability of becoming infected and subsequently infecting other nodes (20).

This study aims to characterize the movement of livestock between districts and evaluate the structure of the livestock trade networks in Uganda using data from the archived AMP booklets. We also discuss the potential impact of such networks on the spread of infectious diseases to inform disease surveillance and control.

## 2. Materials and methods

### 2.1. Data collection and source

**Figure 1:**
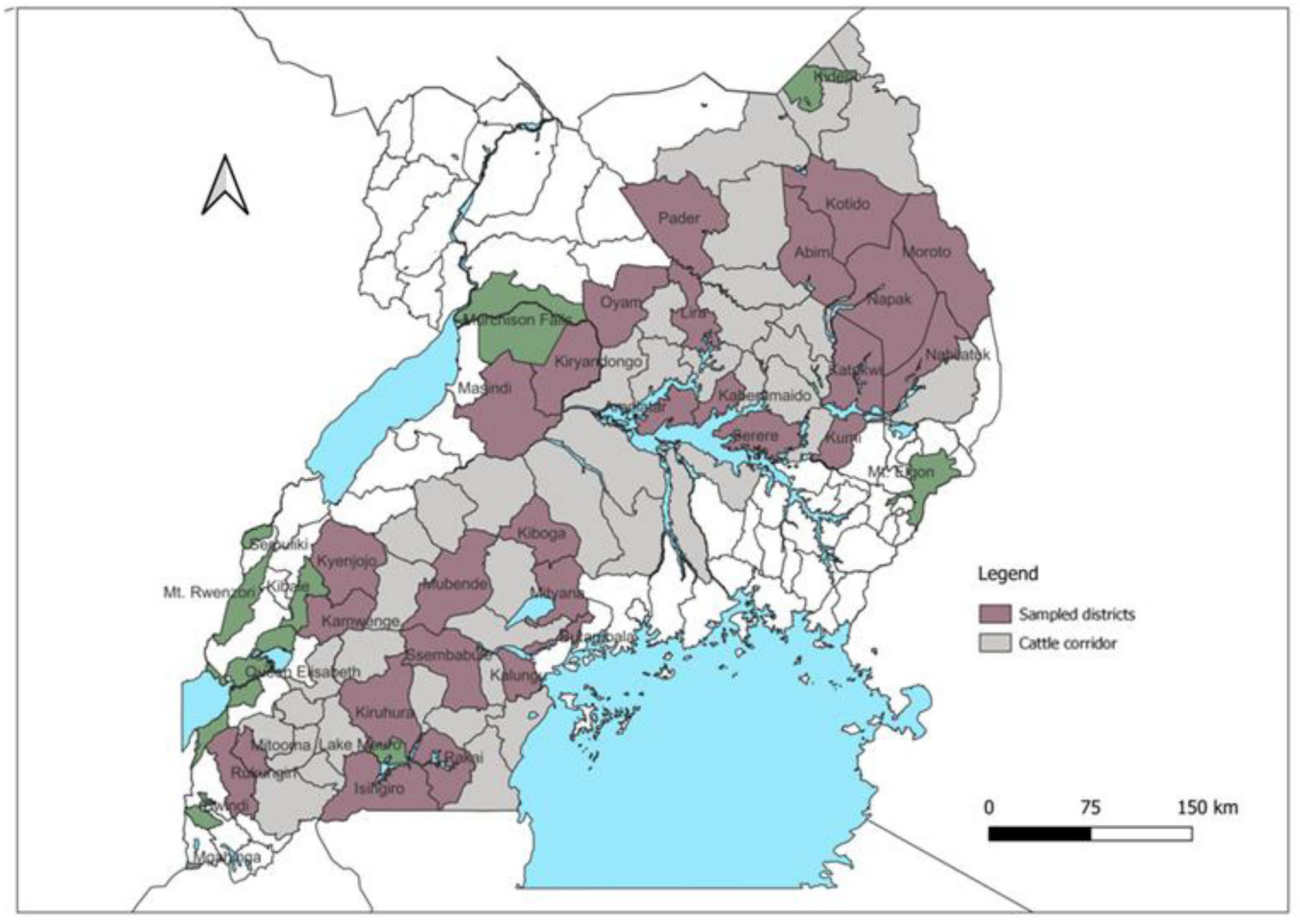
Map of Uganda showing the cattle corridor.

Secondary data in AMPs from the Cattle Corridor were digitized with permission from Uganda's Ministry of Agriculture, Animal Industry and Fisheries (MAAIF). The DVO used the AMPs to permit the movement of animals as well as record the date, number of animals, species, purpose of movement, source, and destination districts of the livestock. The study area, generally referred to as Cattle Corridor stretches diagonally across Uganda, from the southwest to the northeast (Figure 1). It was selected because it is a hotspot for FMD outbreaks (11, 27). The region also has most of the national cattle and small ruminant herds (about 60% of the national herd) (28).

### 2.2. Data entry and management

Information from the 2015 to 2021 (*n* = 18,400) AMP booklets was entered directly into an Excel spreadsheet by eight (8) data clerks and crosschecked by three (3) of the co-authors. The information recorded was: (i) permit number, (ii) date of issuance (i.e., year/month/day), (iii) district of origin, (iv) destination district, (v) species of animal being moved, and (vi) number of animals being moved. Because some districts were missing data in the earlier years, we used data from 2019 to 2021 (*n* = 7,043 APMs).

The data was ordered by year of AMP issuance and district of origin then grouped into 3-month periods to generate movement data by season, i.e., January to March (first dry season), April to June (first wet season), July to September (second dry season) and October to December (second wet season) for each of the years (29). The animal movement data was also grouped by month to generate monthly networks.

### 2.3. Data analysis

#### 2.3.1. Network construction

The networks constructed in this study consisted of nodes, which represent districts animals moved to and from connected by links, which represent the movement of animals between two districts. A district in Uganda is an administrative area averaging 800 km^2^. The nodes (districts) were linked by edges, which were animal movements weighted by number of shipments between the districts per the temporal window the data was grouped by, i.e., monthly, seasonally, or yearly. A shipment event was a batch of one or more animals from a source to a destination district.

Two types of networks were constituted based on the temporal window (monthly: *n* = 30, seasonally: *n* = 10) for each group of animals (cattle, small ruminants and pigs, respectively) using Ucinet6 (Analytical Technologies, USA) (30). The edges between districts in the network were considered static or constant as was in the data and each edge was weighted by the number of direct shipments between the districts. The networks were one-mode type denoting the farm-to-farm direct movement of animals.

We considered seasonal and monthly networks because these allowed us to pinpoint any short-term changes in the network structure, which would be pertinent to the control of a highly infectious disease such as FMD, and equally helpful in understanding the temporal variability in movement patterns. The networks constructed were visualized using Gephi version 0.9.5 (31).

#### 2.3.2. Network analysis

From the networks constructed, we calculated different centrality measures such as in- and out-degree, betweenness and eigenvector values of the nodes. With the centrality measures known, the roles of different nodes in the spread of diseases as a consequence of livestock trade were established. This was critical in identifying nodes for active surveillance, for example in the case of FMD outbreak or as a potential target for strategic vaccinations.

In-degree centrality denoted the number of districts a particular district was connected to by animal purchase while out-degree was determined by the number of districts a particular district sent animals to. On the other hand, betweenness centrality was the frequency with which a district was in the shortest path between pairs of districts in a network. In terms of epizootic control, districts with high betweenness can be critical because they act as conduits that can hasten the spread of a disease to previously unexposed and naïve populations.

We further estimated the network-level characteristics of seasonal and monthly networks for each species by calculating the average path length (APL), average degree, fragmentation, clustering coefficient, density, diameter, and component structure, i.e., the number of components and sizes of the giant strongly or weakly connected components: Giant Strongly Connected Component (GSCC) and Giant Weakly Connected Component (GWCC). The medians were calculated using the “ggplot2” R-package and the figures created using QGIS (32, 33).

We also tested if the generated networks followed small-world and scale-free topologies. A network was considered to have a small-world structure if its clustering coefficient was higher than that calculated from a random network of equivalent size and connections (i.e., with the same number of nodes, edges and density) while its APL was smaller than that of the random network (25, 34). Therefore, to determine if the networks had small-world properties, 100 random networks with the same number of nodes and density as their corresponding empirical networks were generated using Ucinet6. The mean clustering coefficients and average path lengths for the randomly generated networks were then compared with each of their respective opposite empirical networks.

Another useful property of most real-world networks is that the node linkages follow a scale-free power-law distribution. This characteristic is a consequence of two mechanisms: networks spread out continuously by the addition of new nodes, and these new nodes preferring links to other nodes that are already well connected (35). We plotted the fraction of nodes against the in- or out-degree on a logarithmic scale to check if the plots followed the power-law distribution. Spatio-temporal aspects of livestock movements in the cattle corridor were described using tables and graphs.

## 3. Results

### 3.1. Temporal trends of animal movements

**Figure 2:**
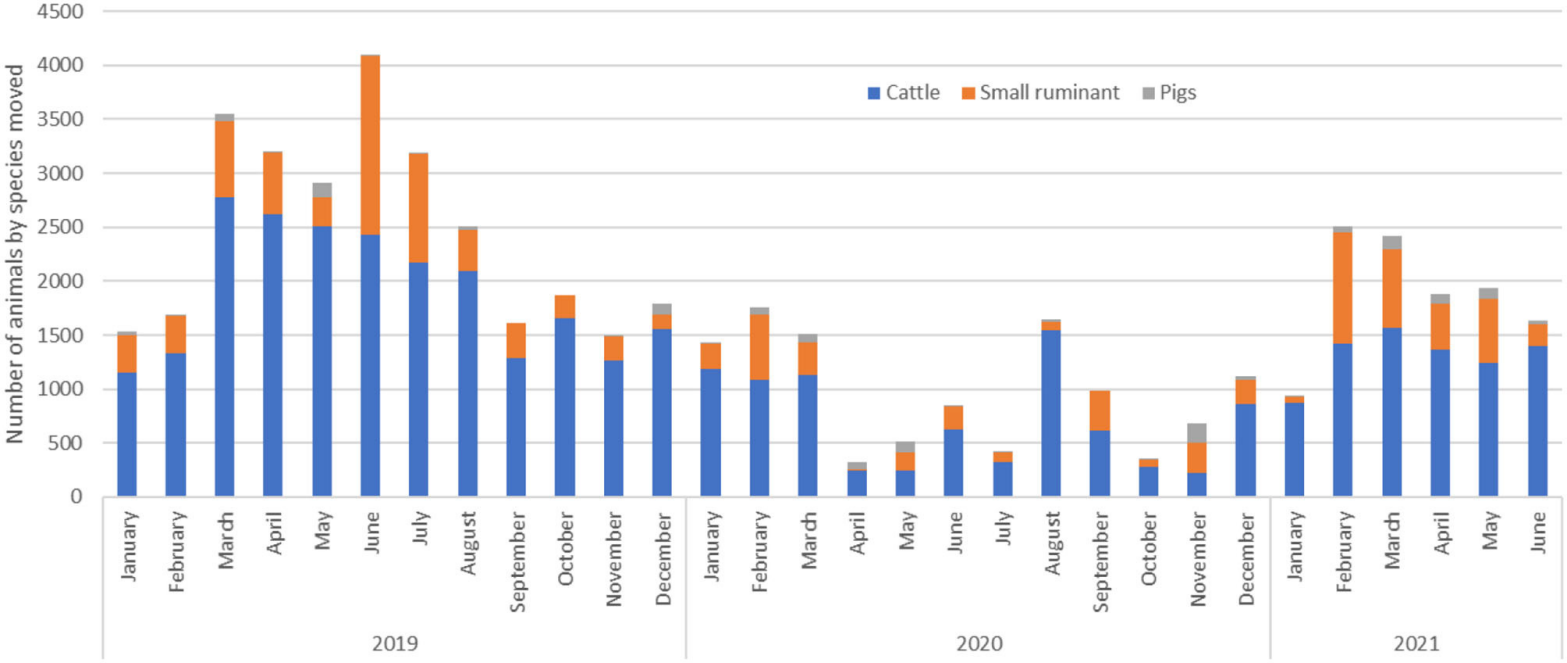
The total number of animals shipped in cattle, small ruminant, and pig monthly movements between trading districts in the Cattle Corridor of Uganda from 2019 to 2021.

Of the 7, 043 AMPs (from 2019 to 2021) used in the study, 87.2% were for cattle movement, 11.2% for small ruminants and 1.6% for pigs. For all species, movements were highest in 2019 before the COVID-19-related lockdown occurred, with the highest volume of animals being traded from March to August 2019. Subsequently, animal movement decreased drastically in 2020 and 2021 (Figure 2). Throughout the study period, the number of cattle traded was twice the number of both small ruminants and pigs. The volume of pigs moved between districts remained steady throughout the study period.

### 3.2. Description of the network structure

The results showed that, across all species, edge densities for seasonal and monthly networks were low, but monthly edge densities were lower than seasonal ones except for the pig networks. All seasonal and monthly networks ranged between 2.4–7 and 2.3–5.2% of the possible edges between nodes across all the species for seasonal and monthly networks, respectively (Table 1). All networks were equally fragmented at seasonal and monthly levels, with an average fragmentation index of 0.9 suggesting a high fraction of isolated pairs of nodes in all the networks.

**Table 1:** Network-level measures for the cattle, small ruminant, and pig seasonal/monthly movements between trading districts in the Cattle Corridor of Uganda from 2019 to 2021.

**Network parameter**	**Mean (minimum, maximum) measure of the network**					
	**Cattle networks**		**Small ruminant networks**		**Pig networks**	
	**Seasonal (*****n*** = **10)**	**Monthly (*****n*** = **30)**	**Seasonal (*****n*** = **10)**	**Monthly (*****n*** = **30)**	**Seasonal (*****n*** = **10)**	**Monthly (*****n*** = **30)**
Density	0.023 (0.019, 0.028)	0.024 (0.017, 0.055)	0.025 (0.017, 0.037)	0.038 (0.02, 0.071)	0.052 (0.014, 0.095)	0.07 (0.04, 0.1)
Nodes	70 (43, 94)	54 (16, 76)	41 (21, 65)	22 (7, 44)	15 (9, 19)	7.4 (5, 11)
Edges	129 (51, 211)	67 (12, 120)	40 (13, 71)	17 (4, 41)	10 (5, 15)	4.7 (3, 8)
Average degree	1.73 (1.19, 2.26)	1.16 (0.74, 1.63)	0.91 (0.57, 1.12)	0.66 (0.14, 0.897)	0.5 (0.1, 0.74)	0.34 (0, 0.7)
Fragmentation	0.9 (0.84, 0.96)	0.92 (0.853, 0.961)	0.89 (0.084, 0.94)	0.89 (0.81, 0.98)	0.9 (0.83, 1)	0.89 (0.83, 0.96)
Average path length	2.56 (1.64, 3.26)	2.29 (1.61, 3.31)	2.78 (1.86, 3.52)	2.22 (1, 3.04)	1.67 (1, 2.5)	1.32 (1, 1.69)
Diameter	6.5 (4, 9)	5.5 (3, 9)	6.5 (4, 9)	5.03 (1, 8)	3.2 (1, 6)	1.82 (1, 3)
Overall clustering coefficient	0.61 (0.16, 1.5)	0.36 (0, 1.4)	0.125 (0, 0.27)	0.076 (0, 0.28)	0 (0, 0)	4.61E + 37 (0, 1E + 38)
Weighted clustering coefficient	0.17 (0.033, 0.37)	0.13 (0, 0.77)	0.067 (0, 0.13)	0.06 (0, 0.23)	0 (0, 0)	0.01 (0, 0.088)
**GWCC**						
Number	6 (4, 10)	8.96 (4, 16)	10.9 (8, 14)	8.24 (4, 15)	6 (3, 10)	5 (3, 10)
Largest size	72.2 (39, 89)	45.25 (11, 71)	30.8 (13, 53)	13.4 (2, 11)	6.3 (1, 15)	3.5 (1, 8)
**GSCC**						
Number	71 (39, 86)	49.38 (15, 71)	37.1 (20, 62)	19.3 (7, 41)	11 (6, 19)	8 (5, 11)
Largest size	7 (1, 15)	4.1 (1, 11)	4.4 (1, 10)	2.9 (1, 7)	1.33 (1, 2)	1.1 (1, 2)

When the direction of the edges was ignored, seasonal networks had more weakly connected components and larger components than monthly networks across all species. In all the seasonal and monthly networks, we found that most of the remaining components contained only a few nodes.

When the direction of the edges was considered, the month with the highest number of components was January 2019 in the bovine networks, with 71 strongly connected components (largest size = 4), while April–June 2019 was the season with the highest number of components, with 86 strongly connected components (largest size = 8) (Table 2).

**Table 2:** Seasons and months that exhibited the highest and lowest numbers of GSCCs and GWCCs by network type for cattle, small ruminant, and pig seasonal/monthly movements between trading districts in the Cattle Corridor of Uganda from 2019 to 2021.

**Network type**	**Period with highest number of GSCCs**	**Period with lowest number of GSCCs**	**Period with highest number of GWCCs**	**Period with lowest number of GWCCs**
Cattle seasonal networks	April–June 2019	April–June 2020	October–December 2020	July–September 2019
Cattle monthly networks	January 2019	April 2020	January 2021	May 2020
Small ruminant seasonal networks	April–June 2019	July–September 2020	January–March 2019	July–September 2020
Small ruminant monthly networks	June 2019	January 2021	March 2019	July 2020
Pig seasonal networks	April–June 2021	July–September 2020	January–March 2020	October–December 2020
Pig monthly networks	May 2021	December 2020	March 2021	June 2021

On average, the APL was shorter in the monthly networks than in seasonal networks across all three species. The monthly pig networks had the shortest mean APL of 1.32 while the seasonal small ruminant network had the highest mean APL (2.78).

The seasonal and monthly networks of cattle and small ruminants followed small-world topologies. The randomly generated networks had lower mean clustering coefficients than the cattle networks for the seasonal and monthly periods at 0.71 and 0.52, respectively. Similarly, the mean APL was much higher for the randomly generated networks than for the seasonal and monthly networks at 6 and 5, respectively. All evaluated pig networks (seasonal and monthly) did not conform to the small-world network topology.

The monthly and seasonal networks for cattle and small ruminants were found to have asymmetric and right-skewed distribution of degrees with long tails, typical degree distributions observed in scale-free networks (35, 36). The monthly and seasonal pig networks did not exhibit typical scale-free characteristics.

### 3.3. Description of node-level metrics

**Figure 3:**
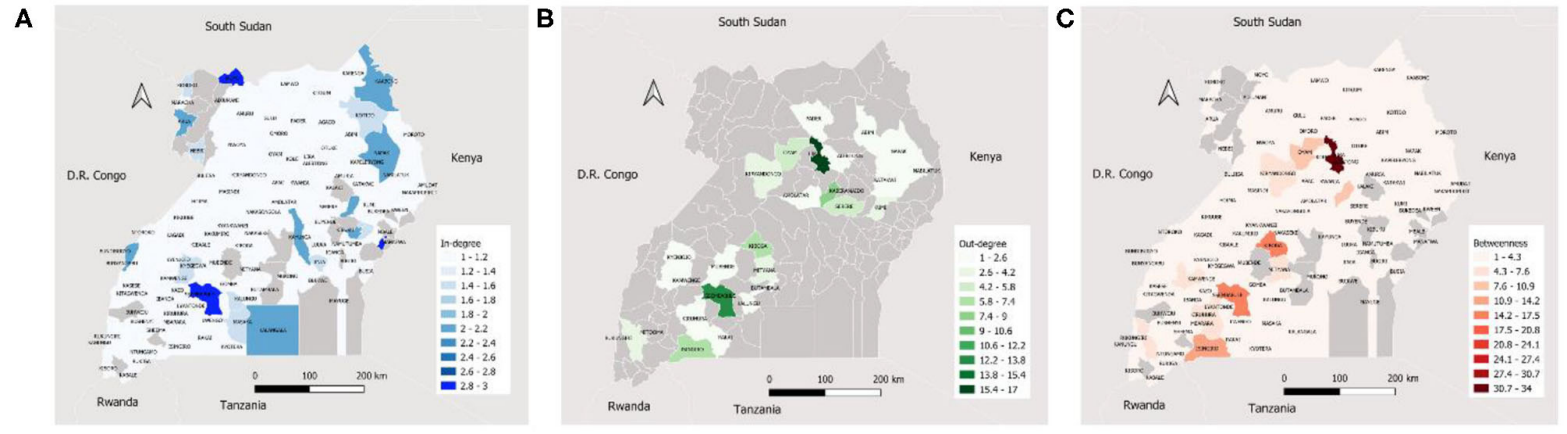
Maps showing the median monthly in-degree **(A)**, out-degree **(B)** and betweenness centrality **(C)** in the cattle inter-district movement networks in the Cattle Corridor of Uganda between 2019 and 2021.

**Figure 4:**
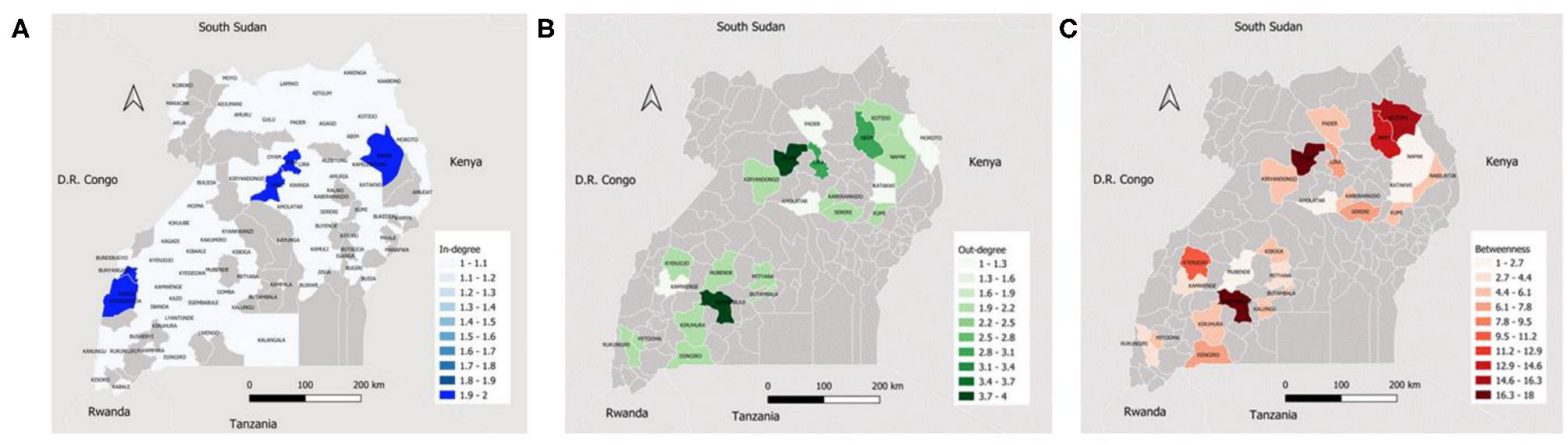
Maps showing the median monthly in-degree **(A)**, out-degree **(B)** and betweenness centrality **(C)** in the small ruminant inter-district movement networks in the Cattle Corridor of Uganda between 2019 and 2021.

**Figure 5:**
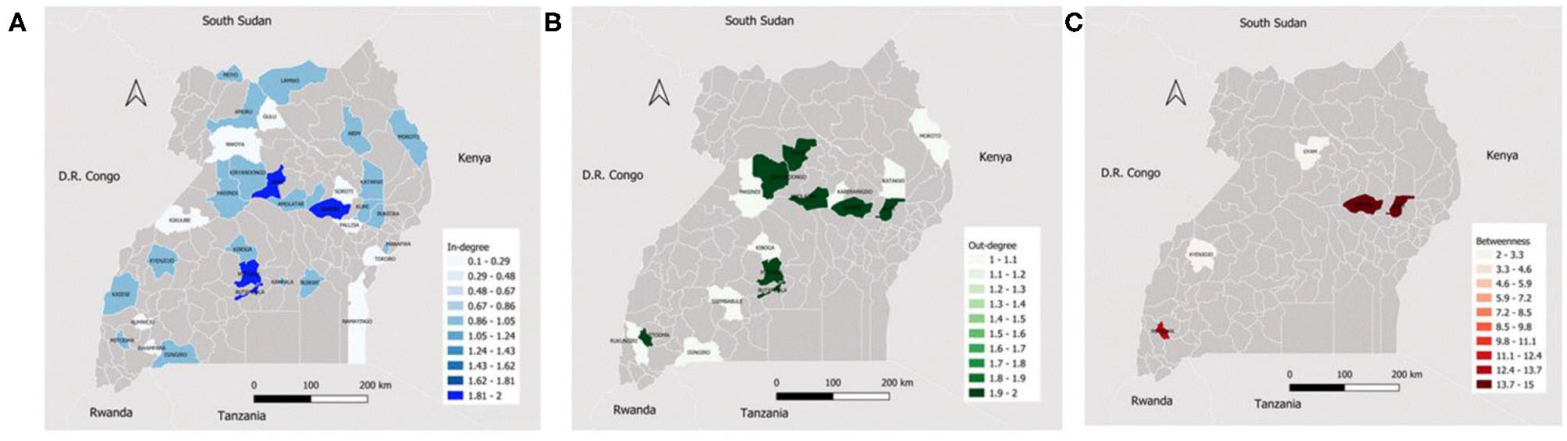
Maps showing the median monthly in-degree **(A)**, out-degree **(B)** and betweenness centrality **(C)** in the pig inter-district movement networks in the Cattle Corridor of Uganda between 2019 and 2021.

Ssembabule District was the only district in the cattle seasonal and monthly networks with both highest median in-degree and out-degree (Figure 3). Lira and Kaberamaido districts showed highest median out-degree for seasonal and monthly cattle networks. In all temporal networks across the species studied, most of the districts that exhibited the highest median in-degree index were bordering one of the five neighboring countries, i.e., Democratic Republic of Congo, Kenya, Tanzania, and South Sudan (Figures 4, 5).

Whereas, Oyam District noticeably had the highest median out-degree across all small ruminant seasonal and monthly networks, Kyenjojo and Butambala districts had the highest median out-degree across all temporal pig networks (Supplementary Figures 1–3). The highest median monthly out-degree (19) was observed in cattle networks in Lira District. The median monthly in-degree ranged from 0–3, 0–1, to 0–1 for cattle, small ruminant, and pig networks, respectively. We also noticed that the districts of Lira, Oyam, and Butambala had the highest betweenness for all network types of cattle, small ruminants, and pigs respectively.

## 4. Discussion

This study was the first of its kind to describe three groups of livestock movements in the Cattle Corridor of Uganda, where we built weighted networks of animal movements. It characterized networks of cattle, small ruminant, and pig movements, which could potentially play a role in the spread of livestock diseases in Uganda.

The seasonal aspect of cattle and small ruminant movement could be related to the fact that from January to March, the Cattle Corridor registered low amounts of rainfall, which translates to shortages of pasture and water, forcing many farmers in the region to depopulate their herds through sale (1). Therefore, many farmers from other regions rush to buy ruminants from the cattle corridor cheaply, resulting in the increased movement of livestock to other districts. This was also observed during another study in the Sahel region, where livestock movements peak prior to the start of the rainy season (37). The additional explanation was that the period between January and March also marks the reopening of schools after the long Christmas holiday. It may suggest that the farmers in the cattle corridor increased sale of animals during this period of the year, especially for small ruminants and pigs to raise money for school fees (38).

The present study revealed the seasonal and monthly livestock trade networks to be compacted networks with many smaller clusters which were intertwined by limited long-distance links. We found that the APL and diameter of the seasonal networks of cattle, small ruminants and pigs were slightly larger in size than their respective monthly networks. Whereas, the small APL and diameter can aid the quick spread of an infectious animal disease to different nodes, the larger APL found in the seasonal networks as compared to the monthly ones could be due to the longer temporal coverage giving rise to chances of exhibiting longer shipments (24).

The small ruminant and cattle networks across the monthly and seasonal timescales had small-world topology. Such networks are prone to facilitating the quick spread of an infectious disease (24, 36). Any infectious agent, once introduced into a small-world network, spreads quickly because of shorter APL between the nodes and higher connectivity among them (34).

Similarly, this study also found that the monthly and seasonal networks for cattle and small ruminant had degree distributions typical of scale-free networks, highlighting heterogeneity in the degree distributions of the districts studied. Such scale-free networks are well-known to facilitate the quick spread of infectious diseases given that they possess hubs with many connections, which once infected can transmit the disease to many nodes quickly (39).

The existence of heterogeneity associated with scale-free networks promotes epidemic spread, not only by surpassing the epidemic threshold, but also by accelerating the propagation of the pathogens within the population (40). For disease preparedness, early warning is paramount in such networks. Strategic nodes (districts) with high in-degree and out-degree could be targeted for surveillance and application of intervention and control measures (24, 34).

Our findings also highlighted the fact that although the geographical adjacency matters in the spread of an infectious disease, even geographically distant nodes can still be connected within a few path lengths which puts them at risk of infectious disease outbreak despite the fact that they are spatially distant (24). This may explain the sporadic outbreaks of FMD in districts which are very distant from the index outbreak districts in Uganda (11). Additionally, when the direction of movements was ignored in the monthly networks, on average, more than 83, 61, and 47.3% of the districts were part of the largest GWCC for cattle, small ruminant, and pig networks, respectively, while a mean of 7.6, 13.2, and 14.9% of the districts were involved in the GSCC for cattle, small ruminant and pig networks. Previous studies have suggested that the GSCC and the GWCC can be taken as indicators of the lower and upper limit of the projected epidemic size, respectively, if there is an outbreak of an infectious disease in a population. Therefore, infectious disease incursions during the months with the highest GSCC and GWCC by species networks would translate into wide transmission (19, 34, 41, 42). Keen interest must be paid to such periods as far as infectious disease surveillance is concerned.

The present study found the border districts of Kasese, Bunyangabu, Bundibugyo, Nebbi, and Arua (which neighbor the Democratic Republic of Congo); Moyo, Kaabong, and Koboko (neighboring South Sudan); Isingiro (touching Tanzania); and Manafwa and Kaabong (bordering Kenya) to have a high in-degree centrality for cattle and small ruminant networks. Whether or not the high number of animal shipments to the border districts could be headed for neighboring countries in undocumented cross-border trade is a detail which this study could not conclude about, but such activity was observed by Lichoti et al. (26) and Mugezi et al. (43) in Uganda. Interestingly, the districts (Lira, Isingiro, Sembabule, Oyam, and Kaberamaido) with highest out-degree have the highest cattle and small ruminant populations in the country (5, 44).

Recent studies showed that higher betweenness nodes were often super-spreaders during the early stages of an outbreak (45–47). Therefore, districts with high betweenness in the cattle (Lira, Insingiro, and Serere), small ruminant (Oyam, Sembabule, and Kiruhura) and pig (Butambala, Kumi, and Serere) networks should be the first targets of intervention during an outbreak to minizine the spread of an infectious disease.

It was noteworthy that the networks based on district-to-district movement of farm animals in the cattle corridor presented very similar structural properties to most other published animal movement networks, even though farming systems were different between countries and production types (15, 21, 34, 48, 49). Although the results did not include all districts in Uganda, they showed the value of such data for epidemiological studies in the country, given that most ruminants are farmed in the cattle corridor. Descriptions of network characteristics as well as network and node-level parameters for different network types obtained from this study can be useful for infectious disease transmission models and for effective management of infectious diseases outbreaks in animals (50).

The biggest limitation of this study was the undocumented inter-district movement of livestock; however, this did not affect the quality of data utilized because these are rare due to the strict policing and could constitute to <4% of livestock movements in the cattle corridor. Another limitation could have been the COVID 19 restrictions which may have affected financially many buyers of cattle.

## 5. Conclusion

Our findings, in the context of low resources, underscored the usefulness of control measures targeting a few “at risk” districts to prevent and contain the spread of infectious diseases effectively. Targeted strategies in the key-player districts identified in this study could mean the following: (i) enhanced bio-security measures, (ii) prioritized active surveillance of selected infectious diseases because of the high risk of infection and spread, and (iii) movement control as an emergency disease control response. We further suggest that a more robust database of intra- and inter-district livestock movements be maintained at all administrative levels, including markets, slaughterhouses, and other gathering points. This could call for the issuance of digital movement permits to ease future network studies and further utilization of the data in preventing the spread of infectious diseases.

## Data availability statement

The raw data supporting the conclusions of this article will be made available by the authors, without undue reservation upon permission from the Ministry of Agriculture, Animal Industry and Fisheries (MAAIF) of Uganda.

## Ethics statement

Ethical review and approval was not required for the animal study because it is the animal movement data.

## Author contributions

Study conception and design: EH, KT, HL, and MD. Data validation: EH, SK, and IM. Data analysis: EH, KT, and HL. Manuscript writing: EH. Manuscript review: EH, KT, HL, MD, and SK. All authors contributed to the article and approved the submitted version.

## Supplementary material

Supplementary Figure 1 Network visualization showing out-degree for **(A)** the most connected seasonal network (April–June 2019) and **(B)** the most connected monthly network (January 2019) in the cattle inter-district movement networks in the Cattle Corridor of Uganda between 2019 and 2021.

Supplementary Figure 2 Network visualization showing out-degree for **(A)** the most connected seasonal network (April–June 2019) and **(B)** the most connected monthly network (June 2019) in the small ruminant inter-district movement networks in the Cattle Corridor of Uganda between 2019 and 2021.

Supplementary Figure 3 Network visualization showing out-degree for **(A)** the most connected seasonal network (April–June 2021) and **(B)** the connected monthly network (May 2021) in the pigs inter-district movement networks in the Cattle Corridor of Uganda between 2019 and 2021.
