# Supplementary figures and images for: Analysis of patterns of livestock movements in the Cattle Corridor of Uganda for risk-based surveillance of infectious diseases
Source: Front Vet Sci. 2023 Jan 23;10:1095293. doi: 10.3389/fvets.2023.1095293 (PMC9899994; doi:10.3389/fvets.2023.1095293)

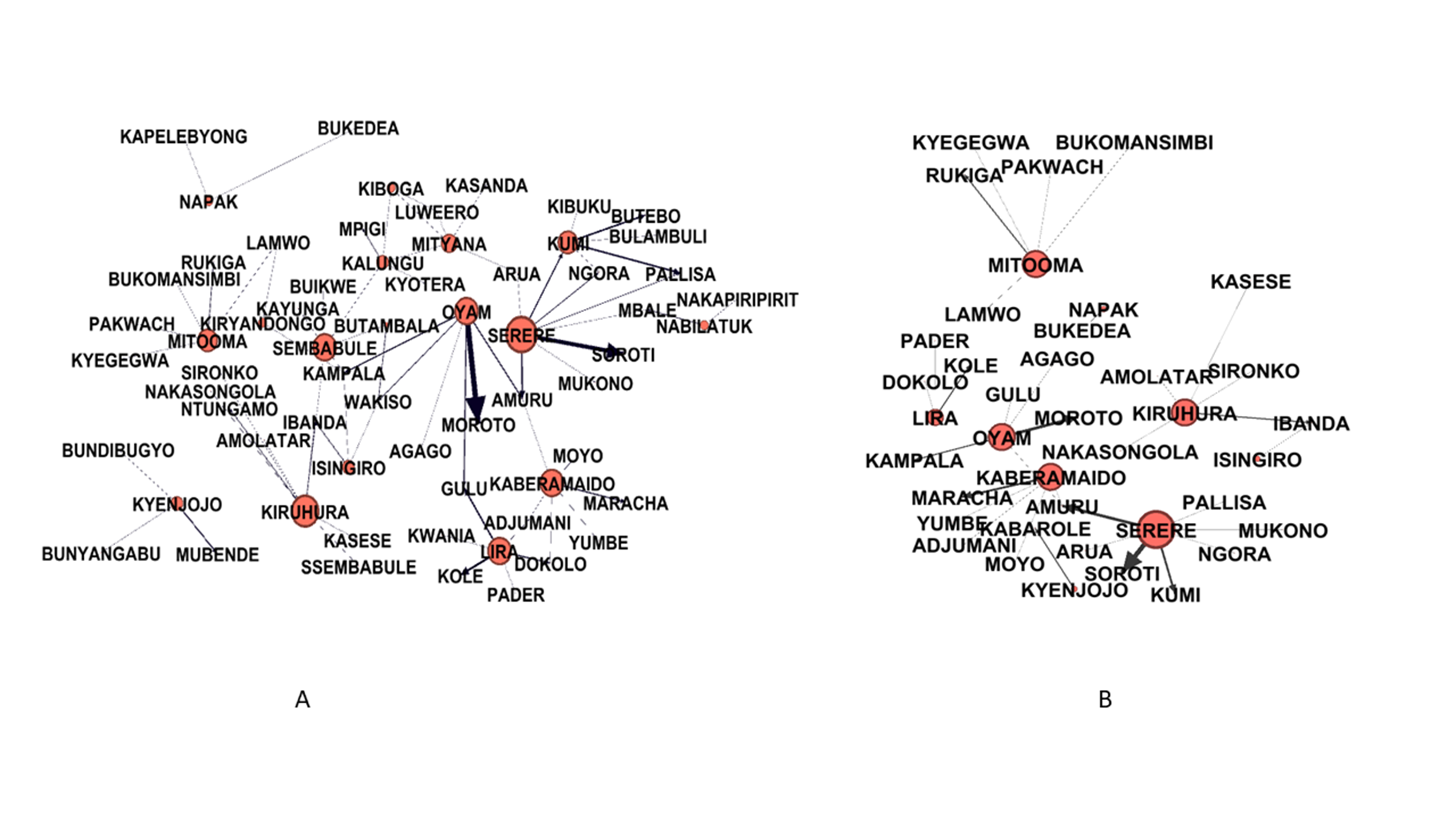

Supplement: Supplementary Figure 1 — Network visualization showing out-degree for (A) the most connected seasonal network (April–June 2019) and (B) the most connected monthly network (January 2019) in the cattle inter-district movement networks in the Cattle Corridor of Uganda between 2019 and 2021. [file Image_1.TIF]

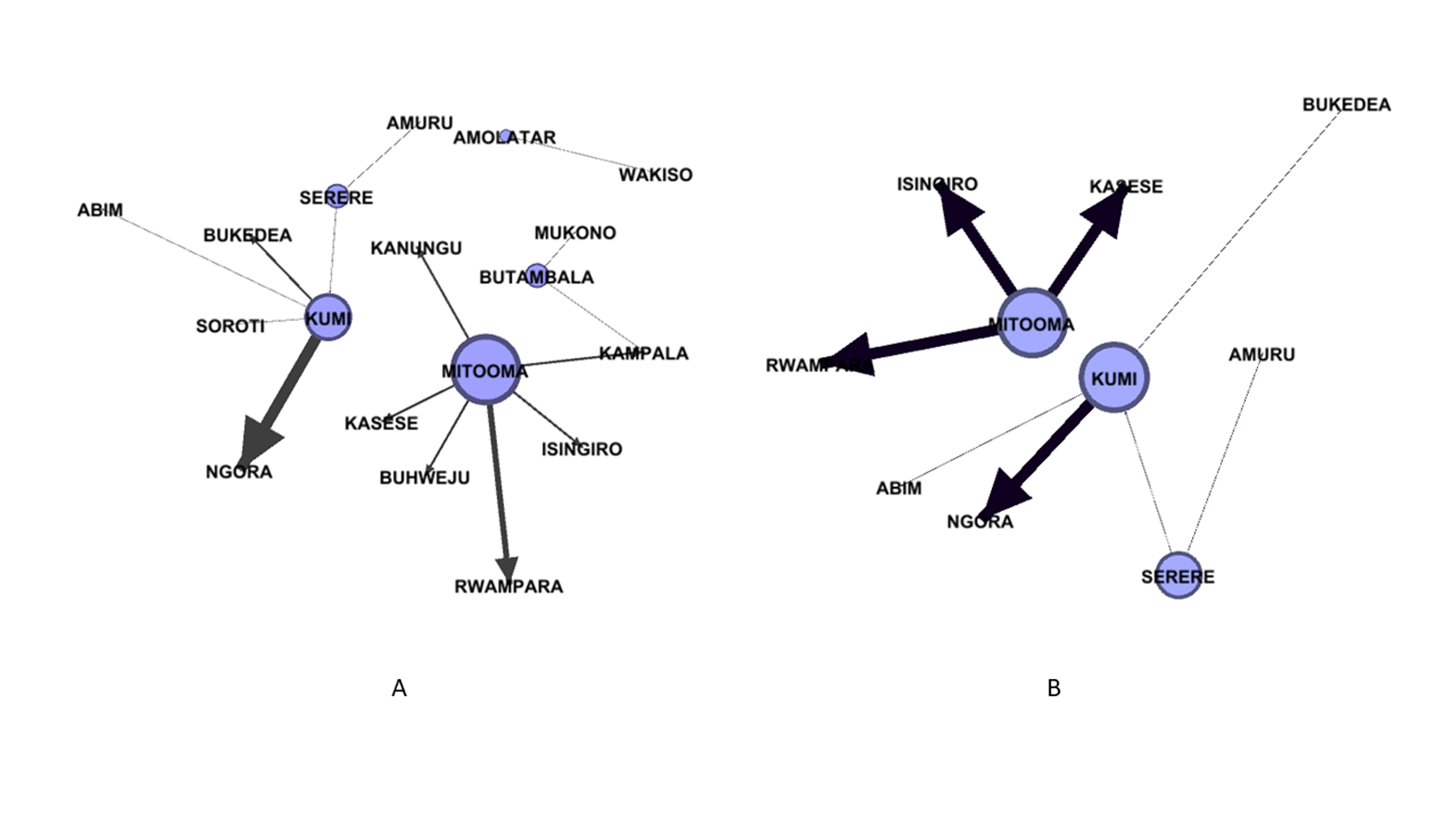

Supplement: Supplementary Figure 2 — Network visualization showing out-degree for (A) the most connected seasonal network (April–June 2019) and (B) the most connected monthly network (June 2019) in the small ruminant inter-district movement networks in the Cattle Corridor of Uganda between 2019 and 2021. [file Image_2.TIF]

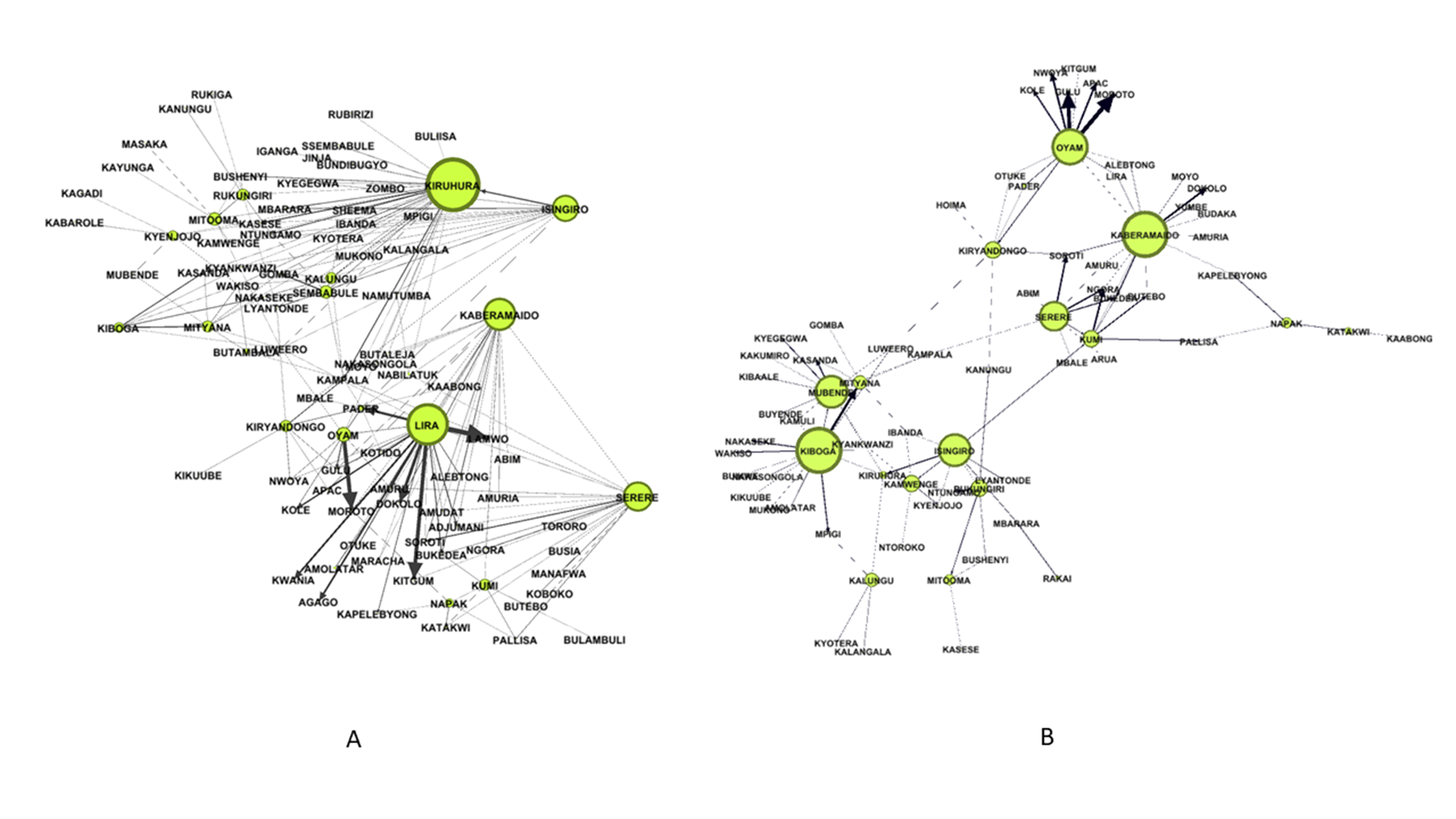

Supplement: Supplementary Figure 3 — Network visualization showing out-degree for (A) the most connected seasonal network (April–June 2021) and (B) the connected monthly network (May 2021) in the pigs inter-district movement networks in the Cattle Corridor of Uganda between 2019 and 2021. [file Image_3.TIF]
